# Supplementary material for: Behavioural and Electrophysiological Responses of Female Anopheles gambiae Mosquitoes to Volatiles from a Mango Bait
Source: J Chem Ecol. 2020 Apr 9;46(4):387–96. doi: 10.1007/s10886-020-01172-8 (PMC7205772; doi:10.1007/s10886-020-01172-8)
Supplement: Supplementary file 2 — (DOCX 313 kb) [file 10886_2020_1172_MOESM2_ESM.docx]

**Behavioural and Electrophysiological Responses of Female *Anopheles gambiae* Mosquitoes to Volatiles from a Mango Bait**

FELICIAN C. MEZA^1,2^, JOE M. ROBERTS^1,3^, ISLAM S. SOBHY^1,4^, FREDROS O. OKUMU^2^, FREDERIC TRIPET^1^, TOBY J.A. BRUCE^1*^

*^1^Centre for Applied Entomology and Parasitology, School of Life Sciences, Huxley Building, Keele University, Keele, Staffordshire, ST5 5BG, UK*

*^2^Environmental Health and Ecological Sciences Department, Ifakara Health Institute, Of Mlabani Passage, P.O. Box 53, Ifakara, Tanzania*

*^3^Centre for Integrated Pest Management, Department of Crop and Environment Sciences, Harper Adams University, Newport, Shropshire, TF10 8NB, UK*

*^4^Department of Plant Protection, Faculty of Agriculture, Suez Canal university, 41522 Ismailia, Egypt*

Felician C. Meza (Orcid ID: 0000-0003-0299-2747)

Joe M. Roberts (Orcid ID: 0000-0002-9576-9239)

Islam S. Sobhy (Orcid ID: 0000-0003-4984-1823)

Toby J.A. Bruce (Orcid ID: 0000-0002-9912-0605)

Frederic Tripet (Orcid ID: 0000-0002-7939-0712)

* *Corresponding author:* Toby J.A. Bruce, [t.j.a.bruce@keele.ac.uk](mailto:t.j.a.bruce@keele.ac.uk)

**Abstract-** Attractive Toxic Sugar Baits (ATSB) are used in a “lure-and-kill” approach for management of the malaria vector *Anopheles gambiae*, but the active chemicals were previously unknown. Here we collected volatiles from a mango, *Mangifera indica*, juice bait which is used in ATSBs in Tanzania and tested mosquito responses. In a Y-tube olfactometer, female mosquitoes were attracted to the mango volatiles collected 24-48 h, 48-72 h and 72-96 h after preparing the bait but volatiles collected at 96-120 h were no longer attractive. Volatile analysis revealed emission of 23 compounds in different chemical classes including alcohols, aldehydes, alkanes, benzenoids, monoterpenes, sesquiterpenes and oxygenated terpenes. Coupled GC-electroantennogram (GC-EAG) recordings from the antennae of *An. gambiae* showed robust responses to 4 compounds: humulene, (*E*)-caryophyllene, terpinolene and myrcene. In olfactometer bioassays, mosquitoes were attracted to humulene and terpinolene. (*E*)-caryophyllene was marginally attractive while myrcene elicited an avoidance response with female mosquitoes. A blend of humulene, (*E*)-caryophyllene and terpinolene was highly attractive to females (*P* < 0.001) when tested against a solvent blank. Furthermore, there was no preference when this synthetic blend was offered as a choice against the natural sample. Our study has identified the key compounds from mango juice baits that attract *An. gambiae* and this information may help to improve the ATSBs currently used against malaria vectors.

**Key words-** Malaria vector, kairomone, attractant, mango, terpenoids

INTRODUCTION

Malaria, primarily vectored in sub-Saharan Africa by the *Anopheles gambiae* Giles (Diptera: Culicidae) mosquito complex, continues to be one of the most important human health issues globally with 219 million cases and 435,000 deaths reported in 2017 alone (World Health Organization, 2018). Reducing incidences of malaria infection relies on controlling the mosquito vectors responsible for transmitting the *Plasmodium* spp. parasites to their human hosts (Mulatier et al. 2019). Key methods for controlling malaria vectoring mosquitoes include insecticide-treated bed nets (ITNs) and indoor residual spraying (IRS) (Bhatt et al. 2015). However, there is increasing evidence suggesting that insecticide resistance is reducing the effectiveness of certain control measures. Thus, controlling malaria vectoring mosquitoes requires new interventions that can work synergistically with existing control tools (Torto 2019). One promising intervention is attractive toxic sugar baits (ATSB), which can be employed for outdoor control, unlike ITNs and IRS, which are primarily developed for indoor use (Adams et al. 2020).

ATSBs exploit mosquito sugar feeding behaviour to lure individuals into a trap treated with a killing agent, such an insecticide (Müller et al. 2008). Both male and female mosquitoes depend on plant sugar, i.e nectar from flowers, sap from leaves and plant stems, to obtain energy for activities such as host-seeking and mating (Foster 1995; Müller and Schlein 2006). This explains why plant volatiles may be attractive to mosquitoes (Nyasembe and Torto, 2014). A growing body of evidence has shown that Afrotropical malaria mosquitoes feed on plant sugars while being found in habitats surrounded by plants (Impoinvil et al. 2004; Manda et al. 2007; Beier et al. 2012). It is thus plausible that that *An. gambiae* females make use of plant odours to for host location (Nyasembe et al. 2012; Nyasembe and Torto, 2014). Nyasembe et al. (2018) have recently shown that *An. gambiae* females can detect plant derived sesquiterpenes and alkenes.

Recently, attractants from fruit juice were used to lure mosquitoes to an insecticide as a development of ATSB (Beier et al. 2012). Tenywa et al. (2017) reported that *Anopheles* spp. mosquitoes were attracted to juice from subtropical fruits such as guava, mango and banana. However, fruit-based attractants used in existing ATSB strategies have a relatively short time period where they are effective as aging and fermentation processes influence their volatile profile (Lebrun et al. 2008; Pandit et al. 2009) and therefore the behavioural response of mosquitoes toward them. An effective long-lasting ATSB strategy would benefit from development of a synthetic semiochemical lure based on the odour of a subtropical fruit known to attract mosquitoes, such as mango, however these attractant chemicals have not yet been identified.

The current study aimed to identify the volatiles from mango juice ATSB that attract *An. gambiae*. To this end, we collected mango volatiles and investigated the behavioural response of *An. gambiae* females to them in a Y-tube olfactometer. Volatile samples were subjected to GC-EAG analysis to determine which compounds elicited electrophysiological responses from the antennae of *An. gambiae* females. Behavioural responses to synthetic compounds were then tested. Identifying chemical attractants that are released from natural fruit juice used in ATSB could help in developing lures which can last longer without deteriorating its active form, in malaria vector monitoring and control programs.

METHODS AND MATERIALS

*Experimental insects*

The Kisumu strain of *Anopheles gambiae sensu stricto* (Giles) (Diptera: Culicidae), colonised from the Kisumu region of Kenya in East Africa, has been maintained at Keele University (UK) in the Centre for Applied Entomology and Parasitology (CAEP) insectaries. Mosquitoes were reared at 27 ± 1 °C and 75 ± 5 % RH with a 12:12 L:D photoperiod. Larvae were fed a diet of ground fish food (Tetramin, Tetra, Melle, Germany) at a rearing density of 200 individuals/litre (Ekechukwu et al. 2015). Pupae were transferred to 5 L plastic cages (20.5 cm height x 20 cm diameter) and covered with netting prior to adult emergence. Approximately 600 - 800 adults were housed per cage. Sugar was provided via a paper towel soaked in 10 % glucose solution and water *via* a soaked cotton pad in an upturned bowl placed on the cage netting. Female adult mosquitoes were fed with defibrinated horse blood (TCS Biosciences, Buckingham, UK) using an artificial feeding membrane (Hemotek Feeding Membrane System, Discovery Workshops, Blackburn, UK). Styrofoam cups containing filter paper and water were placed in the cages four days post blood feeding to collect eggs. Following egg cup removal, the cages were washed thoroughly and sterilised with bleach. Mouth aspirators were used to transfer adults when necessary.

*Volatile collection*

Ripe mango fruits (*Mangifera indica* var. Kent; imported from Senegal) (Tesco, Sutton Coldfield, UK) were washed with distilled water before juice extraction. A 600 ml glass measuring beaker and scalpel was washed with aqueous detergent, rinsed with distilled water and 90 % ethanol (Sigma Aldrich, Gillingham, UK) then dried in a glassware oven at 180 °C for one hour. A single mango fruit was cut into approximately twenty pieces using the scalpel, placed into a clean beaker and blended using a handheld electric blender until homogenised. Distilled water was then added to a total volume of 500 ml. This process was repeated three times with fresh mangoes and clean beakers to give three distinct biological replicates. The blender container, blade and scalpels were washed with aqueous detergent, rinsed with distilled water and 90 % ethanol after each new juice extraction.

For collection of mango juice volatiles, beakers containing 500 ml of mango juice were individually enclosed in a polyethyleneterephthalate oven bag (38 x 25 cm x 12 μm thick; J Sainsbury plc, London, UK) that had been pre-cleaned by heating to 250 °C for one hour (Stewart-Jones and Poppy 2006). Charcoal-filtered air (600 ml/min) was pumped into the bag to maintain positive pressure while air was drawn out (400 ml/min) through a collection filter containing Porapak Q (200 mg, 50-80 mesh; Supelco, Gillingham, UK) held between two silanized glass wool plugs in a disposable glass pipette (4 mm i.d.). Air was circulated through this system using a Pye Volatile Collection Kit (BJ Pye, Hertfordshire, UK). Collections were carried out under laboratory conditions (25 ± 5 °C; 60 ± 10 % RH; 12:12 L:D photoperiod) for five days with the collection filter being replaced every 24 hours to give five samples per mango fruit: 24 h, 48 h, 72 h, 96 h, 120 h. Volatiles were eluted from the Porapak Q filters with diethyl ether (1 x 0.75 ml; 99.7 % purity; Sigma Aldrich, Gillingham UK) and stored at -20 °C until use in bioassays or analysis. The volatile collection process was repeated for each of the three biological replicates.

*Chemical Analysis*

Analyses were carried out on a 7820A GC coupled to a 5977B single quad mass selective detector (Agilent Technologies, Cheadle, UK). The GC was fitted with a non-polar HP5-MS capillary column (30 mm x 0.25 mm x 0.25 µm film thickness) coated with (5%-Phenyl)-methylpolysiloxane (Agilent Technologies) and used hydrogen carrier gas at a constant flow rate of 1.2 ml/min. Automated injections of 1 µl were made using a G4513A autosampler (Agilent Technologies) in splitless mode (285 °C), with oven temperature programmed from 35 °C for 5 min then at 10 °C/min to 285 °C. Compounds were identified according to their mass spectrum, linear retention index relative to retention times of *n*-alkanes, and co-chromatography with authentic compounds.

*Coupled GC-Electrophysiology*

Analysis of collected mango juice volatiles were carried out with a 7820 GC (Agilent Technologies) fitted with flame ionization detector (FID) and a non-polar HP5-MS capillary column (30 mm x 0.25 mm x 0.25 µm film thickness; Agilent Technologies), which used hydrogen carrier gas at a constant flow rate of 1.2 ml/min. Manual injections of 1 µl were in splitless mode (285 °C) with the oven temperature programmed from 35 °C for 5 min then at 10 °C/min to 285 °C. The column effluent was split using a salinized glass push-fit Y-tube connector (Syntech, Kirchzarten, Germany). One arm of this connector was connected with fused silica tubing (50 cm x 0.32 mm i.d.) to the FID (250 °C) and the other to an equal length of deactivated silica tubing passing through a heated (250 °C) transfer line (Syntech) into a glass tube (4 mm i.d.) through which air passed (15 cm/sec) over the EAG preparation.

Electroantennogram recordings were made using an IDAC-2 acquisition controller (Syntech) connected as a second detector of the GC for A/D conversion. Glass electrodes containing electrolyte solution (0.1 M potassium chloride) were attached to silver wires held in micromanipulators (Syntech). Female adult *An. gambiae* were prepared for GC/EAG analysis by excising the head after being chilled in ice for 5 min. The reference electrode was inserted into the back of the head and the circuit was completed by bringing the recording electrode into contact with the tip of one antenna. Both the FID and EAG signals were collected and analysed with GCEAD software (v4.6.1; Syntech). A total of 15 antennae preparations were used for GC/EAG analysis. Volatiles that stimulated responses with at least three different antennae preparations were considered replicable.

*Olfactometer bioassay*

The behavioural responses of female adult *An. gambiae* to volatile chemical stimuli were tested using a Y-tube olfactometer with a 200 mm stem length, 230 mm arm length (60 ° angle) and an internal diameter of 23 mm (Sci-Glass Consultancy, Bere Alston, UK). The olfactometer was placed on a table that was homogeneously illuminated by fluorescent tubes. Airflow in each arm was 100 ml/min and the odour were located at the end of each olfactometer arm. This was similar to the setup used by Peach et al. (2019).

All bioassays were carried out under laboratory conditions (25 ± 5 °C; 60 ± 10 % RH) between 09:00 h and 16:00 h. For all experiments, 4–5-day-old mated female mosquitoes were used, which were sugar-fed with no blood meals. Prior to use in a bioassay, mosquitoes were starved of glucose for a minimum of 24 h. Subsequently, the mosquitoes cage was transferred from the insectary to the olfactometer laboratory for acclimatization one hour before the bioassay. A 10 µl aliquot of headspace sample of mango volatiles, or 10 µl aliquot of test solution (synthetic compounds/blend), was applied to a cut piece of filter paper (6 mm x 15 mm, Whatmann No. 1, GE Healthcare Life Sciences, UK) using a disposable 10 µl glass micropipette (Microcaps, Drummond Scientific Company, USA). Headspace samples and solutions of synthetic compounds were in diethyl ether. The treated piece of filter paper containing test VOCs was then placed at the end of one arm (treated arm), while a filter paper with 10µl of the appropriate solvent control was placed in the other arm (control arm). Individual female mosquitoes were introduced through the stem tube opening using a mouth aspirator and each mosquito was given five minutes to make a choice. Each pair of odour sources was tested either 20 or 40 times with fresh individuals for 5 min (Table S1), and the numbers of mosquitoes reaching the end of each arm during this time was recorded. Mosquitoes that did not make a choice within five minutes after release were considered as non-responding individuals and were excluded from the statistical analysis. To eliminate directional bias, odour source positions were alternated every five releases and new filter papers containing fresh VOC sources were prepared and placed at the end of the olfactometer arms as described above. After each pair of odor sources had been tested five times, glassware was thoroughly cleaned by rinsing with warm water followed by ethanol (Fisher Scientific, Leicestershire, UK) before baking in a glassware oven at 180 °C for 30 min.

*Statistical analyses*

All statistical analyses were performed using R (Version 3.6–1) (R Core Development Team 2019). Y-tube olfactometer bioassay data were analyzed using an exact binomial test against the null hypothesis that the number of mosquitoes reaching the end of either olfactometer arm had a 50:50 distribution. Prior to performing statistical analyses, the replicated results from each of the odor pairs tested were pooled with non-responding individuals being excluded from statistical analyses.

Hierarchical clustering of volatile data over 5 days was visualized using the comprehensive online tool suite MetaboAnalyst 4.0 (Chong et al. 2018). Data matrix was first mean-centered, cube-root transformed prior to analysis. Average linkage hierarchical clustering based on Ward algorithm of the Euclidean distance measure for the differentially released volatiles was used to construct a heatmap.

RESULTS

*Olfactometer bioassay of responses to natural samples*

Female *An. gambiae* were strongly attracted to samples of mango volatiles collected at 24-48h, 48-72h and 72-96h, with at least twice as many mosquitoes choosing the treated arm (Figure 1). Mosquitoes were significantly attracted to mango volatiles when offered a choice compared to a solvent arm (*P* < 0.001 for the 24-48 h sample, *P* = 0.003 for the 48-72 h sample and *P* = 0.016 for the 72-96 h sample). However, volatiles collected at 0-24 h (*P =* 0.065) and 96-120 h (*P =* 0.720) were not attractive.

*Chemical Analysis*

GC-MS Analysis of headspace collections from mango juice revealed the presence of 23 detectable volatiles in 7 chemical classes (alcohols, aldehydes, alkanes, benzenoids, monoterpenes, sesquiterpenes and oxygenated terpenes) at all sampling periods (Table 1). The most abundant compounds were monoterpenes such as 3-carene and α-pinene. A heatmap (Figure S1) shows differential magnitude of volatile emission across collection periods with the highest emission 24-48h sample.

The 24-48h headspace sample of mango volatiles was used for GC-EAG recordings because it was most attractive in bioassays. Four compounds elicited consistent EAG responses with antennae of female *An. gambiae* (Figure 2). These were identified by GC-MS and peak enhancement with co-injection of authentic standards as myrcene, terpinolene, (*E*)-caryophyllene and humulene.

*Olfactometer bioassay of responses to identified compounds*

Two compounds; humulene and terpinolene, elicited a positive behavioural response in the bioassay with female *An. gambiae* ( *P* < 0.001, *P* = 0.039, respectively). Myrcene marginally elicited an avoidance response from mosquito females (*P* = 0.057) whereas (*E*)-caryophyllene marginally attracted them (*P* = 0.063) (Figure 3). As control treatments, citronella was marginally repellent (*P* = 0.057) and mosquito females showed no response (*P* = 1) when given a choice between two arms treated with a solvent blank.

A synthetic blend of humulene, (*E*)-caryophyllene and terpinolene was made up using the same concentration and ratio of compounds as in the 24-48 h natural sample (i.e. 1.9 ng/µl terpinolene + 2.0 ng/µl (*E*)-caryophyllene + 1.6 ng/µl humulene) (Table 1). This synthetic blend was highly attractive to (*P* < 0.001) when tested against a solvent blank and there was no preference when it was offered as a choice against the natural sample (*P* = 1; Figure 4).

DISCUSSION

The current study provides clear evidence of attraction of *An. gambiae* to mango volatiles and identifies the key compounds involved in mediating this behaviour as terpinolene and humulene. These volatiles were attractive both individually and as a blend, also containing (*E*)- caryophyllene, with the same concentration and ratio as the natural sample. In a choice test, there was no distinction between the synthetic blend and the natural sample, showing that the activity of the natural sample was fully accounted for. Although we focused on female insects in the current study, due to their importance as malaria vectors and the need to attract them to bait stations, preliminary experiments showed that *An. gambiae* males were also attracted to the mango volatiles (unpublished data).

Sugar feeding is an important behaviour observed in both male and female mosquitoes that allows them to obtain sufficient energy for physiological processes such as flight, reproduction and adult development (Foster 1995; Manda et al. 2007). These sugar meals are provided by floral and extrafloral nectar or honeydew (Foster 1995; Stone and Foster 2013). Volatile phytochemicals are important olfactory cues used to locate suitable nectar feeding sites by pollinating insects and herbivores (Pichersky and Gershenzon 2002; Bruce et al. 2005). It has been shown that mosquitoes, particularly nocturnal species, make use of the volatiles released by flowering plants (Lahondère et al. 2019; Wondwosen et al. 2017, 2018; Yu et al. 2017) to locate their nectar host plants (Foster and Hancock 1994; Nyasembe and Torto 2014). Zeng et al. (2019) have identified odorant receptors (ORs) from *Culex quinquefasciatus* and *Aedes aegypti* which are sensitive to floral compounds. Moreover, there is increasing evidence that various mosquito species, including *An. gambiae*, show are attracted to certain plants (Gouagna et al. 2010; Manda et al. 2007; Mauer and Rowley 1999; Müller et al. 2011). In addition to other plant parts such as flowers and leaves, female mosquitoes showed an obvious attraction to the odors of fruits (Hien et al. 2016; Müller et al. 2011; Yu et al. 2017) and fruit juices (Tenywa et al. 2017). This is consistent with our results as *An. gambiae* females were significantly attracted to plant volatiles collected from the juice of mango fruits.

Our chemical analysis of mango volatile samples revealed the presence of 23 detectable compounds in seven chemical classes i.e. alcohols, aldehydes, alkanes, benzenoids, monoterpenes, sesquiterpenes and oxygenated terpenes. However, only a subset of these elicited electrophysiological responses with *An. gambiae* antennae. We found that four compounds were consistently detected by the antennae of *An. gambiae*: These were myrcene, terpinolene, (*E*)-caryophyllene and humulene. Previous studies have investigated plant kairomones with mosquitoes. A review by Nyasembe and Torto (2014) reported 29 plant volatile compounds from various chemical classes, including phenols, aldehydes, alcohols, ketones and terpenes that have been identified as mosquito semiochemicals. Nyasembe et al. (2012) documented six EAG-active volatiles for *An. gambiae*; hexanal, β-pinene, limonene and (*E*)-linalool oxide, β-ocimene and (*E*)-β-farnesene. In addition, linalool oxide and linalool were found to evoke strong antennal responses with *C. pipiens* females (Jhumur et al. 2008), suggesting common sensitivity of mosquito females to terpenoids. Earlier studies by Bowen (1992) described two types of broadly- and narrowly-tuned receptor neurones in mosquito antenna sensitive to terpenes and green leaf volatiles. Investigating the antennal recordings of three different mosquito species (i.e. *Aedes aegypti*, *Aedes mcintoshi* and *Aedes ochraceus*), Nyasembe et al. (2018) found that the monoterpenes myrcene and (*E*)-β-ocimene were consistently detected by all the mosquito species in their study. We also recorded an electrophysiological response to myrcene and myrcene was reported earlier as a mango volatile that was EAG active with *Bactrocera dorsalis* fruit flies (Jayanthi et al 2012). Nonetheless, it should be noted that, in addition to terpenoids, aldehydes were also robustly detected by mosquito antenna (Lahondère et al. 2019; Wondwosen et al. 2016, 2017, 2018 ).

Our behavioural results showed that of the four EAG-active volatiles, *An. gambaie* females were attracted to humulene, (*E*)-caryophyllene and terpinolene whereas myrcene elicited an avoidance response. Previous studies have reported attraction but with different compounds. For example, several terpenoids including β-pinene, limonene, (*E*)-β-ocimene and (*E*)-β-farnesene strongly attracted female *An. gambiae* (Nyasembe et al. 2012). Yu et al. (2019) found that volatiles from a nectar host plant, *Abelia chinensis*, mainly composed of aromatics and monoterpenes, were highly attractive to *Culex pipiens pallens* females. Similarly, Otienoburu et al. (2012) found that floral volatiles, mainly aldehydes and terpenoids, from milkweed; benzaldehyde, (*E*)-β-ocimene, phenylacetaldehyde, nonanal, and (*E*)-2-nonenal, elicited attraction of *Culex pipiens* mosquitoes. Interestingly, plant volatiles can be also used as oviposition cues as gravid *An. arabiensis* were attracted to pollen associated volatiles (aldehydes and terpenoids) emitted from surrounding plants which stimulated egg laying (Wondwosen et al. 2016, 2017, 2018).

The plants *Senna didymobotrya* Fresen, *Parthenium hysterophorus* L, *Senna occidentalis* (L.), and *Lantana camara* released attractive volatiles to *An. gambiae*, which primarily consisted of terpenoids (Nikbakhtzadeh et al. 2014). In a dual choice olfactometer, Jacob et al. (2018) showed that a 3-component terpenoid plant-derived blend comprising (*E*)-linalool oxide, β-pinene and β-ocimene was highly attractive to females of *An. gambiae*. Additionally, *Cx. pipiens pallens* females were attracted to terpenoids such as (*E*)-β-ocimene, α-pinene, *β*-pinene, D-limonene and linalool (Yu et al. 2015). Torres-Estrada et al. (2005) identified several compounds from plant extracts, including longifolene and caryophyllene, as attractants for oviposition of *An. albimanus*. It is worth noting that mosquito responses to common plant volatiles is dose-dependent (Hao et al. 2013; Yu et al. 2015). For example, several terpenoids, which were very attractive in our study, showed strong deterrent effects against various mosquito species (Da Silva et al. 2015; Dekker et al. 2011). In other words, lower doses of individual terpenoids elicited an attractive response to mosquito females, while higher doses caused avoidance behaviour (Nyasembe et al. 2012).

We found no distinction between the synthetic blend of attractive terpenoids (*i.e.* humulene, (*E*)-caryophyllene and terpinolene) and the natural sample, indicating that activity of the natural sample could be accounted for by these key compounds. Previous studies have shown the attractiveness of subtractive blends of bioactive compounds derived from full plant volatile profiles to mosquitoes. For example, subtractive synthetic blends of the plant volatiles of *Silene otites* (L.) (acetophenone, linalool oxide, phenyl acetaldehyde and phenylethyl alcohol), milk weed (benzaldehyde, phenylacetaldehyde, and (E)-2- nonenal), maize (benzaldehyde, nonanal, p-cymene, limonene and α-pinene) and rice ((*1R*)-(+) -α -pinene and nonanal), were significantly more attractive when compared with the full volatile blend of these plants (Jhumur et al. 2007; Otienoburu et al. 2012; Wondwosen et al. 2017, 2018, respectively).

Our study has identified the key compounds in mango juice baits that are responsible for attraction of *An. gambiae* mosquitoes. Natural extracts currently used in ATSB traps, as we have shown, lose their attractiveness after 4 days. The attractive 3-component blend of mango terpenoids could be used to develop a synthetic semiochemical lure for long-lasting outdoor monitoring and control of the malaria vector *An. gambiae*. However, while the current results are promising, field and semi-field studies, optimizing the efficiency of terpenoid-baited traps, are still required before upscaling its application in controlling malaria vector mosquito and we plan to conduct such experiments in future research. The olfactometer bioassay was small scale. Background odors from naturally occurring vegetation hosts may reduce the attractiveness of the terpenoid blend in outdoor complex environments. Furthermore, mosquitoes in the field will have varying physiological condition and exist as different strains or even species. Our findings contribute to the understanding of mosquito attraction to plant odours and identify candidate chemical compounds from which to develop a synthetic semiochemical lure based on mango fruit for use in ATSB control strategies.

ACKNOWLEDGEMENTS

This work was supported by a Wellcome Trust Fellowship to F Meza (Grant Number: 208322/Z/17/Z). Special thanks to Simon Ashall for rearing mosquitoes. FM would like to thank Dr. Fredros Okumu and Dr. Sarah Moore from Ifakara Health Institute for their continuous support. Keele University receives grant aided support from the BBSRC.

REFERENCES

Adams F, Adams JECF, Camara S, et al (2020) Indoor use of attractive toxic sugar bait in combination with long ‑ lasting insecticidal net against pyrethroid ‑ resistant *Anopheles gambiae* : an experimental hut trial in Mbé , central Côte d ’ Ivoire. Malar J 19:11. doi: 10.1186/s12936-019-3095-1

Beier JC, Müller GC, Gu W, et al (2012) Attractive toxic sugar bait (ATSB) methods decimate populations of *Anopheles* malaria vectors in arid environments regardless of the local availability of favoured sugar-source blossoms. Malar J 11:31. doi: 10.1186/1475-2875-11-31

Bhatt S, Weiss DJ, Cameron E, et al (2015) The effect of malaria control on *Plasmodium falciparum* in Africa between 2000 and 2015. Nature 526:207–211. doi: 10.1038/nature15535

Bowen MF (1992) Terpene-sensitive receptors in female *Culex pipiens* mosquitoes: Electrophysiology and behaviour. J Insect Physiol 38:759–764. doi: 10.1016/0022-1910(92)90028-C

Chong J, Soufan O, Li C, et al (2018) MetaboAnalyst 4.0: Towards more transparent and integrative metabolomics analysis. Nucleic Acids Res 46:W486–W494. doi: 10.1093/nar/gky310

Da Silva RCS, Milet-Pinheiro P, Da Silva PCB, et al (2015) (*E*)-Caryophyllene and α-humulene: *Aedes aegypti* oviposition deterrents elucidated by gas chromatography-electrophysiological assay of commiphora leptophloeos leaf oil. PLoS One 10:e0144586. doi: 10.1371/journal.pone.0144586

Dekker T, Ignell R, Ghebru M, et al (2011) Identification of mosquito repellent odours from *Ocimum forskolei*. Parasites and Vectors 4:183. doi: 10.1186/1756-3305-4-183

Ekechukwu NE, Baeshen R, Traorè SF, et al (2015) Heterosis increases fertility, fecundity, and survival of laboratory-produced F1 hybrid males of the malaria mosquito *Anopheles coluzzii*. G3 Genes, Genomes, Genet 5:2693–2709. doi: 10.1534/g3.115.021436

Foster WA (1995) Mosquito sugar feeding and reproductive energetics. Annu Rev Entomol 40:443–474. doi: 10.1146/annurev.ento.40.1.443

Foster WA, Hancock RG (1994) Nectar-related olfactory and visual attractants for mosquitoes. J Am Mosq Control Assoc 10:288–96

Gouagna LC, Poueme RS, Dabiré KR, et al (2010) Patterns of sugar feeding and host plant preferences in adult males of *An. gambiae* (Diptera: Culicidae). J Vector Ecol 35:267–276. doi: 10.1111/j.1948-7134.2010.00082.x

Hao H, Sun J, Dai J (2013) Dose-dependent behavioral response of the mosquito *Aedes albopictus* to floral odorous compounds. J Insect Sci 13:27. doi: 10.1673/031.013.12701

Hien DF d. S, Dabiré KR, Roche B, et al (2016) Plant-mediated effects on mosquito capacity to transmit human malaria. PLoS Pathog 12:e1005773. doi: 10.1371/journal.ppat.1005773

Impoinvil DE, Kongere JO, Foster WA, et al (2004) Feeding and survival of the malaria vector gambiae on plants growing in Kenya. Med Vet Entomol 18:108–115. doi: 10.1111/j.0269-283X.2004.00484.x

Jacob JW, Tchouassi DP, Lagat ZO, et al (2018) Independent and interactive effect of plant- and mammalian- based odors on the response of the malaria vector, *Anopheles gambiae*. Acta Trop 185:98–106. doi: 10.1016/j.actatropica.2018.04.027

Jhumur US, Dötterl S, Jürgens A (2008) Floral odors of *Silene otites*: Their variability and attractiveness to mosquitoes. J Chem Ecol 34:14–25. doi: 10.1007/s10886-007-9392-0

Jhumur US, Dötterl S, Jürgens A (2007) Electrophysiological and behavioural responses of mosquitoes to volatiles of *Silene otites* (Caryophyllaceae). Arthropod Plant Interact 1:245–254. doi: 10.1007/s11829-007-9022-3

Lahondère C, Vinauger C, Okubo RP, et al (2019) The olfactory basis of orchid pollination by mosquitoes. bioRxiv 643510. doi: 10.1101/643510

Lebrun M, Plotto A, Goodner K, et al (2008) Discrimination of mango fruit maturity by volatiles using the electronic nose and gas chromatography. Postharvest Biol Technol 48:122–131. doi: 10.1016/J.POSTHARVBIO.2007.09.010

Manda H, Gouagna LC, Foster WA, et al (2007) Effect of discriminative plant-sugar feeding on the survival and fecundity of *Anopheles gambiae*. Malar J 6:113. doi: 10.1186/1475-2875-6-113

Mauer DJ, Rowley WA (1999) Attraction of *Culex pipiens* pipiens (Diptera: Culicidae) to flower volatiles. J Med Entomol 36:503–507. doi: 10.1093/jmedent/36.4.503

Mulatier M, Camara S, Koffi A, et al (2019) Efficacy of vector control tools against malaria-infected mosquitoes. Sci Rep 9:6664. doi: 10.1038/s41598-019-43195-6

Müller G, Schlein Y (2006) Sugar questing mosquitoes in arid areas gather on scarce blossoms that can be used for control. Int J Parasitol 36:1077–1080. doi: 10.1016/j.ijpara.2006.06.008

Müller GC, Kravchenko VD, Schlein Y (2008) Decline of *Anopheles sergentii* and *Aedes caspius* populations following presentation of attractive toxic (Spinosad) sugar bait stations in an oasis. J Am Mosq Control Assoc 24:147–149. doi: 10.2987/8756-971x(2008)24[147:doasaa]2.0.co;2

Müller GC, Xue R De, Schlein Y (2011) Differential attraction of *Aedes albopictus* in the field to flowers, fruits and honeydew. Acta Trop 118:45–49. doi: 10.1016/j.actatropica.2011.01.009

Nikbakhtzadeh MR, Terbot JW, Otienoburu PE, Foster WA (2014) Olfactory basis of floral preference of the malaria vector *Anopheles gambiae* (Diptera: Culicidae) among common African plants. J Vector Ecol 39:372–383. doi: 10.1111/jvec.12113

Nyasembe VO, Tchouassi DP, Pirk CWW, et al (2018) Host plant forensics and olfactory-based detection in Afro-tropical mosquito disease vectors. PLoS Negl Trop Dis 12:e0006185. doi: 10.1371/journal.pntd.0006185

Nyasembe VO, Teal PE, Mukabana WR, et al (2012) Behavioural response of the malaria vector *Anopheles gambiae* to host plant volatiles and synthetic blends. Parasites and Vectors 5:1–11. doi: 10.1186/1756-3305-5-234

Nyasembe VO, Torto B (2014) Volatile phytochemicals as mosquito semiochemicals. Phytochem Lett 8:196–201. doi: 10.1016/j.phytol.2013.10.003

Otienoburu PE, Ebrahimi B, Phelan PL, Foster WA (2012) Analysis and optimization of a synthetic milkweed floral attractant for mosquitoes. J Chem Ecol 38:873–881. doi: 10.1007/s10886-012-0150-6

Pandit SS, Kulkarni RS, Chidley HG, et al (2009) Changes in volatile composition during fruit development and ripening of ‘Alphonso’ mango. J Sci Food Agric 89:2071–2081. doi: 10.1002/jsfa.3692

Peach DAH, Gries R, Young N, et al (2019) Attraction of female *Aedes Aegypti* (L.) to aphid honeydew. Insects 10:43. doi: 10.3390/insects10020043

Pichersky E, Gershenzon J (2002) The formation and function of plant volatiles: perfumes for pollinator attraction and defense. Curr Opin Plant Biol 5:237–243. doi: 10.1016/S1369-5266(02)00251-0

R Core Development Team (2019) R: A language and environment for statistical computing. In: Cowles RS, Soana CR, Holdcraft R, Loeb GM (eds) R Foundation for Statistical Computing. Vienna

Stewart-Jones A, Poppy GM (2006) Comparison of glass vessels and plastic bags for enclosing living plant parts for headspace analysis. J Chem Ecol 32:845–864. doi: 10.1007/s10886-006-9039-6

Stone CM, Foster WA (2013) Plant-sugar feeding and vectorial capacity. In: Takken W, Koenraadt CJM (eds) Ecology of parasite-vector interactions, Ecology and control of vector-borne diseases. Wageningen Academic, pp 35–79

Tenywa FC, Kambagha A, Saddler A, Maia MF (2017) The development of an ivermectin-based attractive toxic sugar bait (ATSB) to target *Anopheles arabiensis*. Malar J 16:338. doi: 10.1186/s12936-017-1994-6

Torres-Estrada JL, Meza-Alvarez RA, Cibrian-Tovar J, et al (2005) Vegetation-derived cues for the selection of oviposition substrates by *Anopheles albimanus* under laboratory conditions. J Am Mosq Control Assoc 21:344–349. doi: 10.2987/8756-971x(2006)21[344:vcftso]2.0.co;2

Torto B (2019) Innovative approaches to exploit host plant metabolites in malaria control. Pest Manag Sci. doi: 10.1002/ps.5460

Wondwosen B, Birgersson G, Seyoum E, et al (2016) Rice volatiles lure gravid malaria mosquitoes, *Anopheles arabiensis*. Sci Rep 6:37930. doi: 10.1038/srep37930

Wondwosen B, Birgersson G, Tekie H, et al (2018) Sweet attraction: Sugarcane pollen-associated volatiles attract gravid *Anopheles arabiensis*. Malar J 17:1–9. doi: 10.1186/s12936-018-2245-1

Wondwosen B, Hill SR, Birgersson G, et al (2017) A(maize)ing attraction: gravid *Anopheles arabiensis* are attracted and oviposit in response to maize pollen odours. Malar J 16:1–9. doi: 10.1186/s12936-016-1656-0

World Health Organization (2018) WHO | The World malaria report 2018. World Health Organization

Yu BT, Ding YM, Hu Y, et al (2019) Attraction of *Culex pipiens* pallens (Diptera: Culicidae) to floret volatiles and synthetic blends of its nectar host plant *Abelia chinensis* (Rubiales: Caprifoliaceae). J Med Entomol 56:29–34. doi: 10.1093/jme/tjy161

Yu BT, Ding YM, Mo JC (2015) Behavioural response of female *Culex pipiens* pallens to common host plant volatiles and synthetic blends. Parasites and Vectors 8:1–8. doi: 10.1186/s13071-015-1212-8

Yu BT, Huang SQ, Ding YM, et al (2017) Laboratory evaluation of differential attraction of *Culex pipiens* pallens to the volatiles of flowers, fruits, and seed pods. J Asia Pac Entomol 20:1372–1376. doi: 10.1016/j.aspen.2017.06.010

TABLES

**Table 1.** Emission (ng) (mean ± SE; *n* = 3) of volatile organic compounds from mango (*Mangifera indica* var. Kent) juice entrained for 5 days in periods of 24h.

| **Volatile compounds^*^** | **RI** | **Entrainment period** | | | | |
| --- | --- | --- | --- | --- | --- | --- |
|  |  | 0-24 h | 24-48 h | 48–72 h | 72-96 h | 96–120 h |
| **Alcohols** |  |  |  |  |  |  |
| (*E*)-3-hexen-1-ol | 863 | 1.30±0.64 | 0.88±0.26 | 0.35±0.11 | 0.78±0.42 | 0.61±0.12 |
| (*E*)-2-Octen-1-ol | 980 | ND | 0.06±0.04 | 0.15±0.03 | 0.81±0.51 | 0.16±0.03 |
| Phenylethyl alcohol | 1136 | ND | 0.45±0.33 | 0.49±0.03 | 0.99±0.50 | 1.55±0.50 |
| p-Cymen-7-ol | 1380 | 1.16±0.79 | 0.51±0.37 | 0.14±0.06 | 1.80±0.75 | 2.92±1.11 |
| **Aldehydes** |  |  |  |  |  |  |
| (Z)-6-Nonenal | 1294 | 0.08±0.07 | 0.37±0.21 | 0.08±0.06 | 0.24±0.19 | 1.73±0.65 |
| **Alkenes** |  |  |  |  |  |  |
| 1-Decene | 1088 | 0.23±0.14 | 0.16±0.06 | 0.11±0.04 | 0.24±0.08 | 0.30±0.13 |
| **Benzenoids** |  |  |  |  |  |  |
| Indole | 1351 | 0.24±0.15 | 0.97±0.56 | 0.79±0.24 | 0.85±.20 | 0.69±0.24 |
| **Monoterpenes** |  |  |  |  |  |  |
| *α*-Pinene | 933 | 0.55±0.05 | 0.53±0.07 | 0.47±0.02 | 0.29±0.08 | 0.41±0.04 |
| β-Myrcene | 992 | 0.91±0.19 | 1.23±0.42 | 1.06±0.33 | 0.75±0.26 | 1.13±0.12 |
| α-Phellandrene | 1002 | 0.39±0.05 | 0.52±0.16 | 0.33±0.08 | 0.27±0.08 | 0.32±0.02 |
| 3-Carene | 1008 | 36.01±3.42 | 47.58±12.09 | 36.04±8.28 | 23.57±10.31 | 34.23±1.25 |
| α-Terpinene | 1015 | 0.24±0.03 | 0.29±0.10 | 0.15±0.06 | 0.13±0.06 | 0.13±0.06 |
| p-Cymene | 1024 | 0.36±0.07 | 0.41±0.12 | 0.27±0.07 | 0.21±0.03 | 0.42±0.07 |
| D-Limonene | 1028 | 1.24±0.12 | 1.71±0.51 | 1.34±0.33 | 0.88±0.38 | 1.33±0.03 |
| Terpinolene | 1112 | 1.40±0.18 | 1.99±0.78 | 0.94±0.51 | 0.85±0.35 | 1.04±0.07 |
| **Sesquiterpenes** |  |  |  |  |  |  |
| α-copaene | 1396 | 0.54±0.32 | 0.99±0.71 | 0.23±0.07 | 0.43±0.12 | 0.48±0.17 |
| β-Elemene | 1411 | 0.29±0.14 | 0.34±0.13 | 0.24±0.09 | 0.16±0.07 | 0.27±0.05 |
| α-Gurjunene | 1415 | 0.47±0.13 | 0.54±0.22 | 0.47±0.09 | 0.31±0.08 | 0.35±0.01 |
| (*E*)-caryophyllene | 1425 | 2.37±1.12 | 2.04±0.81 | 2.49±1.06 | 0.66±0.23 | 1.72±0.51 |
| Humulene | 1460 | 1.74± 0.91 | 1.59±0.72 | 1.65±0.72 | 0.54±0.05 | 1.31±0.38 |
| δ-Cadinene | 1529 | 0.19±0.06 | 0.32±0.11 | 0.24±0.03 | 0.12±0.05 | 0.13±0.05 |
| **Oxygenated terpenes** |  |  |  |  |  |  |
| (*E*)-Limonene oxide | 1166 | 0.13±0.04 | 0.18±0.09 | 0.11±0.01 | 0.08±0.03 | 0.18±0.04 |
| Caryophyllene oxide | 1591 | 0.21±0.08 | 0.25±0.11 | 0.18±0.05 | 0.07±0.02 | 0.29±0.08 |

Under each chemical class, volatiles are ordered in accordance with their increasing retention time in a gas chromatograph.

* Volatiles were tentatively identified with spectra and high-probability matches (>85%) according to NIST mass spectral database. EAG active compounds were confirmed by coinjection with authentic standards.

RI: Retention indices were calculated from retention times relative to a series of n-alkanes (C8-C20) analysed on a HP-5 column.

The shaded rows represent the volatiles that possess electrophysiological activities to *Anopheles gambiae* females.

ND= not detected.

FIGURE LEGENDS

**Fig. 1** Behavioural response of *Anopheles gambiae* naïve females in a two-choice Y-olfactometer (percentage attracted, *n* = 40). Mosquitoes were given the choice between two odours: Control = Diethyl ether as solvent control; Treatment = Mango (*Mangifera indica* var. Kent) juice headspace sample of volatiles entrained for 5 days in periods of 24h*.* Mango volatiles were dissolved using diethyl ether. Numbers in parentheses inside each bar represent the total number of mosquitos that chose each olfactometer arm. Both percentages and absolute numbers (in parentheses) of nonresponding mosquitos are presented on the right-hand side (‘no choice’). Asterisks indicate a preference that was signiﬁcantly different (binomial test) from a 50:50 distribution: **P* < 0.05; ****P* < 0.001; NS not significant. Nonresponding mosquitos were excluded from the statistical analysis.

**Fig. 2** Coupled GC-EAG analysis showing antennal response of female *Anopheles gambiae* to volatiles collected from Mango (*Mangifera indica* var. Kent) juice. Upper trace = antennal response, lower trace = FID response. The EAG-active volatiles for *A. gambiae* were identified as: (1) myrcene; (2) terpinolene; (3) (*E*)-caryophyllene and (4) humulene.

**Fig. 3** Behavioural response of *Anopheles gambiae* naïve females in a two-choice Y-olfactometer (percentage attracted, *n* = 20). Mosquitoes were given the choice between two odours. EAG active compounds were tested against diethyl ether as solvent control. Compounds tested were: (1) myrcene, (2) terpinolene, (3) caryophyllene and (4) humulene. Two additional control treatments, (5) diethyl ether and (6) citronella, were also tested. Numbers in parentheses inside each bar represent the total number of mosquitos that chose each olfactometer arm. Both percentages and absolute numbers (in parentheses) of nonresponding mosquitos are presented on the right-hand side (‘no choice’). Asterisks indicate a preference that was signiﬁcantly different (binomial test) from a 50:50 distribution: **P* < 0.05; NS not significant. Nonresponding mosquitos were excluded from the statistical analysis.

**Fig. 4** Behavioural response of *Anopheles gambiae* naïve females in a two-choice Y-olfactometer (percentage attracted, *n* = 40). Mosquitoes were given the choice between two odours. The synthetic blend contained three attractive EAG active volatiles (terpinolene, (*E*)-caryophyllene and humulene) using the same concentration and ratio of compounds as in the 24-48 h natural sample dissolved in diethyl ether (DEE). Natural blend was the whole blend of mango volatiles collected at 24-48 h. The bioassay was carried out by releasing 40 adult females individually at the base of a two-choice Y-olfactometer and evaluating their response 5 min after their release or after the first choice was made. Numbers in parentheses inside each bar represent the total number of mosquitos that chose each olfactometer arm. Both percentages and absolute numbers (in parentheses) of nonresponding mosquitos are presented on the right-hand side (‘no choice’). Asterisks indicate a preference that was signiﬁcantly different (binomial test) from a 50:50 distribution: ****P* < 0.001; NS not significant. Nonresponding mosquitos were excluded from the statistical analysis.
